# Improving the Flame Retardance of Polyisocyanurate Foams by Dibenzo[d,f][1,3,2]dioxaphosphepine 6-Oxide-Containing Additives

**DOI:** 10.3390/polym11081242

**Published:** 2019-07-26

**Authors:** Johannes Lenz, Doris Pospiech, Maxime Paven, Rolf W. Albach, Martin Günther, Bernhard Schartel, Brigitte Voit

**Affiliations:** 1 Leibniz-Institut für Polymerforschung Dresden e.V., Hohe Str. 6, 01069 Dresden, Germany; 2 Organic Chemistry of Polymers, Technische Universität Dresden, 01062 Dresden, Germany; 3 Covestro Deutschland AG, 51365 Leverkusen, Germany; 4 Bundesanstalt für Materialforschung und-prüfung (BAM), 12205 Berlin, Germany

**Keywords:** flame retardant, dibenzo[d,f][1,3,2]dioxaphosphepine 6-oxide, DOPO, phospha-Michael addition, polyisocyanurate

## Abstract

A series of new flame retardants (FR) based on dibenzo[d,f][1,3,2]dioxaphosphepine 6-oxide (BPPO) incorporating acrylates and benzoquinone were developed previously. In this study, we examine the fire behavior of the new flame retardants in polyisocyanurate (PIR) foams. The foam characteristics, thermal decomposition, and fire behavior are investigated. The fire properties of the foams containing BPPO-based derivatives were found to depend on the chemical structure of the substituents. We also compare our results to state-of-the-art non-halogenated FR such as triphenylphosphate and chemically similar phosphinate, i.e. 9,10-dihydro-9-oxa-10- phosphaphenanthrene-10-oxide (DOPO), based derivatives to discuss the role of the phosphorus oxidation state.

## 1. Introduction

Polyurethanes that are synthesized by polyaddition of polyisocyanates and polyols to generate a class of materials with an extremely adaptable property profiles [1]. The molar excess of the polyisocyanate results in crosslinked polyisocyanurate (PIR) structures. The isocyanurate rings increase the stability of the material as compared to the corresponding polyurethanes [2,3,4,5]. Furthermore, crosslinked PIR foams do not melt. Both crosslinking and isocyanurate rings result in enhanced char formation after burning. Therefore, these properties as well as their closed cell structure, excellent strength at low density, and low thermal conductivity PIR foams are particularly well-suited as insulation materials [1] e.g., as insulation panels in constructions. However, a need for reduction of energy costs requires more efficient insulation, and recent incidents underline the importance of efficient fire protection in building and construction [1,6,7]. Of particular importance is the formation of insulating char on the surface of buildings to protect the structures from excessive heat.

For PIR, additive tris(2-chloro isopropyl)phosphate (TCPP) is the state-of-the-art flame retardant (FR) [8,9]. It combines both halogen and phosphate in one molecule and it leads to a good balance in gas phase versus solid phase FR action in the resulting foams. TCPP also lowers the viscosity of the formulation for improved processing and plasticizes the foam [10]. However, there is interest in halogen-free FRs to comply with certain eco-labels, for instance the label “pure life” [11]. Phosphorus (P)-containing molecules are considered to be substitutes for halogenated FRs [9,12,13]. Phosphorus offers a variety of structures with different oxidation states of the phosphorus atom [14]. Generally speaking, the mode of action, i.e., gas phase versus solid phase action, is dominated by two aspects for phosphorous based flame retardants [2,15,16,17,18]. First, the oxidation state of the phosphorus atom in the compounds plays a decisive role. If the phosphorus atom has a high oxidation state a thermally stable char is more likely to be formed and the emission of P-containing gases (gas phase mechanism) is low. If the phosphorus oxidation state is low, P-containing gases are more likely to be released. Gas phase action is strongest for phosphine oxides and low for phosphonates [16]. Secondly, the volatility of the FR impacts the gas phase activity [19]. Balancing gas phase and condensed phase activity in polymer materials is important to tune FR performance and comply with safety standards [17,18,20].

Isobutyl bis(hydroxymethyl) phosphine oxide with an oxidation state of P of –I is an example of an FR agent with high gas phase activity [17]. Phosphinates, e.g., aluminum diethyl phosphinate [18,21], 9,10-dihydro-9-oxy-10-phosphaphenanthrene-10-oxide (DOPO), and its derivatives [13,22,23] have an oxidation state of +I and they are well-known FR additives for many polymeric materials. DOPO derivatives (either as free, non-reactive additive, or incorporated into a polymer backbone) mostly introduce a combination of gas phase/condensed phase action and intumescence [24,25,26]. Derivative incorporating secondary amines yielded phosphonamidates that were applied as FR in polyurethanes [27]. Phosphonates with intermediate oxidation state of +III, e.g., diethyl ethylphosphonate (DEEP) and dimethyl phenylphosphonate (DMPP) have been established as halogen-free FRs for polyurethanes, polyesters, and polyamides [28]. In all cases, the P-containing compounds were applied as non-reactive FR. DOPO [29], phosphates [20,30], and phosphinic oxide compounds [17,31] can also be used to prepare P-containing diols that are employed as reactive FRs in polyurethane formulations.

**Figure 1:**
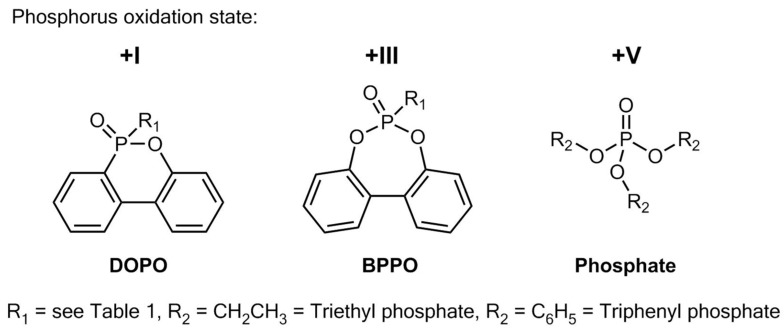
Chemical structures of 9,10-dihydro-9-oxy-10-phosphaphenanthrene-10-oxide (DOPO), dibenzo[d,f][1,3,2] dioxaphosphepine 6-oxide (BPPO), and the descibed phosphates with the assigned oxidation state of the phosphorus atom.

Here, we explore the efficiency of dibenzo[d, f][1, 3, 2] dioxaphosphepine 6-oxide (BPPO) compounds as FR in PIR foams. The BPPO compounds are phosphonates with an oxidation state of +III and are compared to the DOPO compounds with an oxidation state of +I. Triphenyl phosphate (TPP), a state-of-the-art non halogenated FR with an oxidation state of +V, served as benchmark. The structural motives are presented in Figure 1. Triethyl phosphate (TEP), a common additive in PIR foams, was also part of most formulations. TEP acts as FR but also plastics the material, improving mechanical properties of the PIR foams. This study aims to investigate differences in phosphonate, phosphinate and phosphate (TPP) based FR in technically relevant PIR formulations.

The synthesis of the novel BPPO compounds that are listed in Table 1 has been previously reported by our group [32]. We found that the synthesis of BPPO proceeds under significantly milder conditions than those reported for DOPO [33]. 

The addition of BPPO to unsaturated compounds via a Phospha-Michael addition [23,34] more readily proceeds and the products are easier to purify. The FR efficiency of the new BPPO compounds are compared in rigid PIR foams with DOPO compounds and with benchmark foam with TEP and TPP. The formulations were kept constant according to Table 2, and only the amount of FR was varied to maintain a constant P-content to ensure the comparability of the foams. The stepwise variation of the chemical structure of the FR provides the opportunity to assign the occurring effects to structural features.

## 2. Materials and Methods

### 2.1. Chemicals

Pentane (≥99%, Sigma-Aldrich, Darmstadt, Germany); triphenyl phosphate (TPP, >99.0%, TCI, Eschborn, Germany), triethyl phosphate (TEP, >99.0%, TCI, Eschborn, Germany); PEG 400 (Sigma-Aldrich, Darmstadt, Germany), nOH = 280 mg KOH/g; potassium acetate (KAc, ≥99.0%, Sigma-Aldrich, Darmstadt, Germany); diethylene glycol (DEG, >99.0%, Sigma-Aldrich, Darmstadt, Germany); phthalate/DEG polyester polyol PEP50AD (Covestro Deutschland AG, Dormagen, Germany), nOH = 240 mg KOH/g; polymeric isocyanate DESMODUR 44V70L, NCO% = 30.9 (Covestro Deutschland AG, Dormagen, Germany); Emulsogen TS100 (Clariant, Pratteln, Switzerland), polyether modified polysiloxane TEGOSTAB B 8421, nOH = 57 mg KOH/g (Evonik Industries, Darmstadt, Germany); p-tolyl isocyanate (99%, Sigma-Aldrich, Darmstadt, Germany), 1,4-dioxane (99.8%, Sigma-Aldrich, Darmstadt, Germany) were used as received. For PIR foam preparation, a solution of 25 wt% potassium acetate in DEG (nOH: 793 mg KOH/g) was used as a catalyst. The synthesis of the BPPO-containing FRs according to Table 1 has been recently described [32]. The DOPO-derivative of dimethyl itaconate (DMI-DOPO) was synthesized according to Pospiech et al. [35].

### 2.2. Foam Preparation

The preparation of the PIR foams was performed in two steps. First, a mechanical stirrer mixed the polyester polyol, stabilizers, surfactants, catalyst, blowing agent, and FR at 2000 rpm. Subsequently, the required amount of isocyanate was added. After mixing for a few seconds, the mixture was poured into an open mold to prepare the foam. During the foaming process, three distinct times (cream time, setting time, and rise time) were noted all starting from the mixing of polyol formulation and isocyanate. Cream time is defined as the point at which the mixture turns creamy and starts to expand. The setting time, which is also called gel time or fiber time, reflects the time when solid fibers can be drawn from the expanding foam. The rise time indicates when the foam expansion is completed. After the foam expanded, it was removed from the mold and kept at room temperature for 24 h before the specimens were cut with a band-saw. 

Table 2 summarizes the general composition of the foams. The complete formulations are listed in Table S1 in the Supplementary Materials. 

### 2.3. Characterization of Foam Properties

The density, pore sizes, and cell integrity of the foams were characterized. The samples used for cone calorimetry (10 cm × 10 cm × 5 cm) were employed to calculate the density and their volume and weight was determined. Pore sizes were determined from light microscopic images. The microscope used was an Axio Imager (ZEISS) that was equipped with Axiocam 305 color camera (ZEISS, Oberkochen, Germany).

The scanning electron microscopy (SEM) images were taken by a Gemini Ultra plus SEM (ZEISS, Oberkochen, Germany). The water absorption was determined as a parameter for the cell integrity. Foam cubes of 4 cm × 4 cm × 4 cm were completely immersed in boiling water for 90 min. The mass difference (*m_w_* − *m_d_*) was determined (*m_w_* describes the mass of the wet foam, *m_d_* mass of the dry foam). With the density of water (1 g·cm^−3^), the mass difference was divided by the sample volume (*a*^3^) and normalized to 100%. With the following Equation (1), the water absorption *WA_V_* (volume of absorbed water over foam volume) was obtained.

(1) $$WA_{V}=\frac{m_{w}-m_{d}}{a^{3}\times \rho }\times 100\%$$

#### 2.3.1. Mechanical Properties of the Foams

The compressive strength of the foams was investigated by a TIRAtest 2300 instrument (TIRA GmbH, Schmalkalden, Germany). The foams were tested according to ISO 604 while using a 100 kN force sensor. A traverse was used for the position sensor. An initial force of 2 N was applied and the test was conducted with a velocity of 500 mm·min^−1^. The test specimen cubes with edge lengths of 5 cm were used. The compression curves were perpendicularly measured to the foam rise direction.

#### 2.3.2. ATR-FTIR Analysis

The infrared spectra were measured with a Vertex 80v spectrophotometer (Bruker, Rheinstetten, Germany) equipped with a golden gate diamond ATR unit (SPECAC) in the wavenumber range of 4000–600 cm^−1^ with 100 scans per measurement. A MCT (Mercury cadmium telluride, Bruker, Rheinstetten, Germany) detector was used with a resolution of 4 cm^−1^.

#### 2.3.3. Quantitative Phosphorus Content

The quantitative P-content was determined by Mikroanalytisches Labor Kolbe (Mülheim a.d. Ruhr, Germany). 

#### 2.3.4. Thermal Decomposition

The thermal behavior was assessed by thermogravimetric analysis (TGA) using a TGA Q500 (TA Instruments, New Castle, UK) in nitrogen atmosphere (60 mL⋅min^-1^) from 25 to 800 °C at a scan rate of 10 K⋅min^-1^ with a sample weight of 5 mg. The thermal decomposition products were analyzed with pyrolysis-GC/MS. These experiments were carried out with a GC 5890 (Agilent Technologies, Santa Clara, USA) coupled with a pyroprobe 2000 (CDS Instruments, Oxford, USA) under helium atmosphere and a flow rate of 1.0 mL·min^−1^.

### 2.4. Fire behavior

#### 2.4.1. Vertical Flame Spread

The vertical flame spread (VFS) test was carried out according to DIN 4102 (B2 classification). Thus, samples with dimensions of 20 cm × 10 cm × 1 cm were vertically hung in the test chamber (here, a test chamber for UL-94 test was used). A burner flame was applied for 15 s on the lower edge of the specimen. The height of the flame was measured. The test was passed when the height of the flame was lower than or equal to 15 cm.

#### 2.4.2. Cone Calorimeter

The prepared PIR foams were cut into specimens with dimensions of 10 cm × 10 cm × 5 cm. The samples were conditioned for 48 h at 23 °C in a climate chamber with 50% relative humidity. They were tested in a cone calorimeter after 48 h (Fire Testing Technology, East Grinstead, UK) according to ISO 5660-1:1990. The heat flux was adjusted to 50 kW·m^−2^. The distance of the burner to sample was 25 mm. Aluminum foil was wrapped around the sides of the probe to avoid edge burning. During the measurement, time to ignition (t_ig_), heat release rate (HRR), peak of heat release rate (PHRR), time to PHRR (tPHRR), maximum of the average rate of heat emission (MARHE), total heat released (THR), total mass loss (TML), and effective heat of combustion (EHC, as calculated by THR/TML) were evaluated.

## 3. Results and Discussion

### 3.1. Foam Compositions

**Figure 2:**
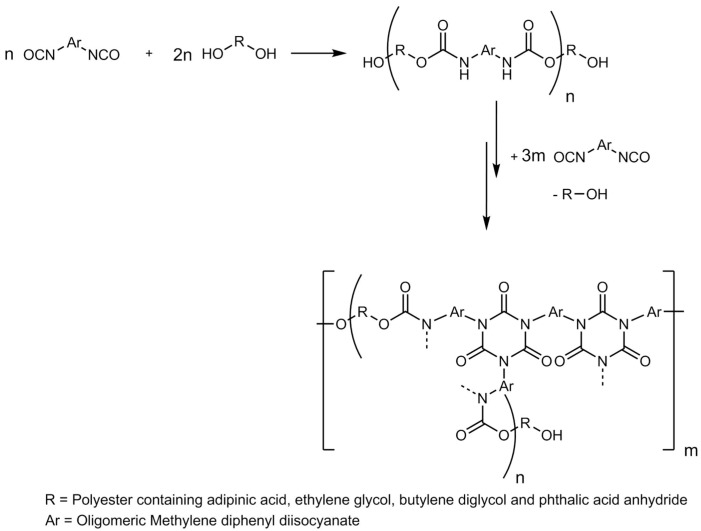
Formation of the polyurethane/polyisocyanurate (PUR/PIR) structures in the foams with the used formulation.

The foams were prepared with compositions according to Table 2. An NCO/OH ratio (molar ratio) of 3.2 (NCO index 320) was maintained for all the foams. At this index, the foams contain a mixture of urethane and isocyanurate structures (Figure 2). For simplicity, the foams are referred to as PIR foams.

In these PIR foams, the chemistry of the FR additive was varied. The foams Ref-0%P, TEP-0.3%P, TPP-0.7%P, and TPP/TEP-1.0%P were used as the control foams. The formulation of Ref-0%P was used as reference and did not contain any phosphorus. In TEP-0.3%P and in most of the other foams, TEP was added at a concentration of 0.3 wt.% phosphorus to lower the polyol viscosity and improve the mixing with the isocyanate. In general, TEP increases the activity of the acetate catalyst. Foam TPP/TEP-1.0%P with TEP and TPP with a P-content of 1 wt.% was used as the benchmark. In the following, TPP was replaced by BPPO- and DOPO-based derivatives incorporating acrylates and benzoquinone. Table 1 provides the systematic structural variation. In case of the acrylate derivatives, the structure of the substituent was varied (methyl, ethyl, t-butyl, and phenyl). These compounds can be used as non-reactive flame retarding additives. BPPO was added to acrylamide and p-benzoquinone, with the intention of yielding additives reactive in the PIR foam. For comparison, all of the corresponding DOPO-Phospha-Michael adducts were synthesized. However, only DMI-DOPO and benzoquinone derivative, hydroquinone-DOPO (HQ-DOPO), could be isolated in reasonable purity and were compared to the respective BPPO derivatives.

The foams were analyzed by ATR-FTIR spectroscopy. The FTIR spectra of the foams are shown in the Supporting Information (Figures S1 and S2). The spectrum of the pure EA-BPPO (Figure S3 in the Supplementary Materials) showed intense bands at 717 (P-C stretching), 925 (P-Ar), and 1242 cm^−1^ (P=O stretching) [36]. In the FTIR spectrum of foam EA-BPPO/TEP-1.0%P with 7 wt.% EA-BPPO (matching 1 wt.% phosphorus in the foam) the same bands appeared with weaker intensity. The stretching vibration bands for urethane at 1218 cm^−1^ and isocyanurate at 1405 cm^−1^ indicated the successful reaction of the isocyanate groups with aliphatic OH groups of the polyol [37]. A residue of non-reacted isocyanate groups was observed at 2273 cm^−1^. This is due to the high NCO index of 3.2 that is necessary for generating isocyanurate rings [37].

In the case of AM-BPPO, HQ-BPPO, and HP-BPPO, a reaction between isocyanate and amide groups (AM-BPPO) and aromatic OH groups (HQ-BPPO, HP-BPPO), respectively, was assumed to occur during foam generation. These reactions could not be proven by FTIR. Therefore, model reactions between p-tolyl isocyanate and FR in stoichiometric amounts catalyzed by potassium acetate were performed in DMSO-d6 at 80 °C (a temperature that can be detected during foam preparation inside the material). After one hour, ^1^H NMR indicated a reaction of HP-BPPO with the isocyanate. A reaction between AM-BPPO and isocyanate to acetylurea could not be proven. Consequently, it was concluded that HP-BPPO and HQ-BPPO belong to the group of reactive FRs [17]. The stability of the BPPO ring against OH groups was examined with EA-BPPO in ethanol in the presence of potassium acetate at 80 °C for 1 h. EA-BPPO was found to be stable under these conditions, as the ^1^H NMR only showed the educts and no degradation products. The spectrum of EA-BPPO was preserved (as compared to NMR spectrum in [32]). Therefore, it was expected that the BPPO ring is stable in the foaming process. The BPPO compounds were subjected to transesterification with diols (ethylene glycol, 1,4-butane diol) catalyzed by Ti(OBu)_4_ to obtain reactive FRs (Table S2 and Table S3). A reaction could not be observed under various conditions, in most cases the BPPO ring opened. For SU-BPPO and DMI-BPPO, the synthesis of oligomers with different diols, as outlined before for DOPO derivatives [38], was also examined (Table S2). These experiments were not successful and they showed ring opening of the BPPO phosphonate ring.

### 3.2. Foam Properties

The foams were prepared under comparable reaction conditions (see experimental section). The foam morphology, density, water uptake, and compression strength of the various samples were analyzed to investigate the influence of the composition on the physical properties. Table 3 summarizes the results. The sample names contain the type of FR as well as the total P-content in wt.%.

The P-contents of the foams that are listed in Table 3 were calculated from the starting foam formulation taking the contribution of all phosphorus compounds into account. In the foams with EA-BPPO, MA-BPPO, AM-BPPO, and HQ-BPPO, the content of FR was varied to achieve P-contents between 1.0 to 1.5 wt.%. The quantitative P-contents were analyzed by Mikroanalytisches Labor Kolbe (Germany) for selected samples (Table S5). The contents found were slightly higher (1.1 wt.% for TPP/TEP-1.0%P and 1.3 wt.% for EA-BPPO/TEP-1.0%P) than the calculated equivalents (1.0 wt.%).

The control foams Ref-0%P and TEP-0.3%P had densities in the range of 36 kg·m^−3^. The addition of TPP resulted in a slight increase to 39 kg·m^−3^. The BPPO compounds added at a concentration of 1 wt.% phosphorus yielded foams in the desired density range of 38–42 kg·m^−3^. DMI-DOPO presented a comparable result. This density is typical for foams with steel facings that are used in cold store construction. Higher BPPO FR contents increased the density, as in the case of DMI-DOPO. Foams that were prepared with AM-BPPO and HQ-BPPO showed densities of around 50 kg·m^−3^. This is attributed to the fact that kinetic parameters of foam preparation (cream time, setting time, rise time) were significantly lower than for all other foams. The BPPO additives, except DMI-BPPO, were powders that were milled to comparable particle size (50–100 μm) prior to use. It was noted that all BPPO compounds, except AM-BPPO, SU-BPPO, and HQ-BPPO, were soluble in the formulation at elevated temperature.

**Figure 3:**
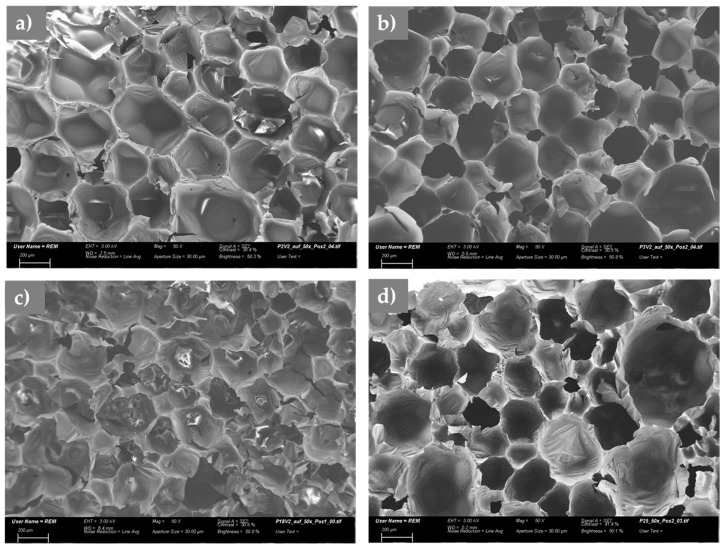
Examples of SEM images of foam (**a**) TEP-0.3%P; (**b**) TPP/TEP-1.0%P; (**c**) MA-BPPO/TEP-1.0%P; and (**d**) AM-BPPO/TEP-1.3%P, scale bar: 200 μm.

The foam appearance that was observed in SEM was independent of the solubility of the FR. In all cases, homogeneous foams without visible particles (note the starting particle size of 50–100 μm) within the foam cells were obtained. Figure 3 shows SEM images of foams TEP-0.3%P (Figure 3a), TPP/TEP-1.0%P (Figure 3b), MA-BPPO/TEP-1.0%P (with soluble MA-BPPO, Figure 3c), and AM-BPPO/TEP-1.3%P (with insoluble AM-BPPO Figure 3d) as examples. The pore sizes of the foams according to optical microscopy varied between 0.14 and 0.20 mm. The differences between the foams were not significant.

The water uptake *WA_V_* is a measure for cell integrity (open or partially perforated cell windows). The values that are summarized in Table 3 indicate different degrees of cell integrity. The foams with EA-BPPO, MA-BPPO, and HQ-BPPO did not show a change in cell integrity when compared to the control and benchmark foams. All other foams had much larger contents of window perforation.

**Figure 4:**
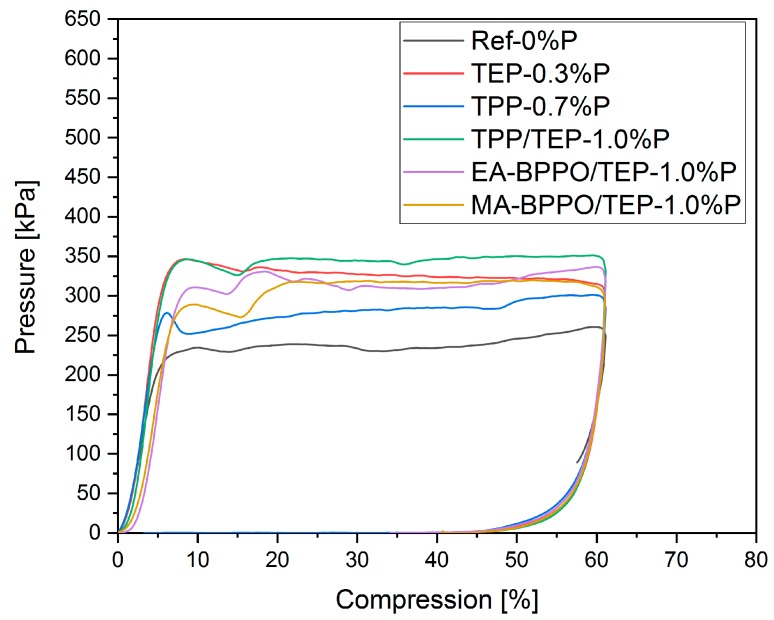
Pressure compression diagram of selected foams.

The mechanical behavior of the selected foams was examined by the compression test until a maximum compression of 60% (Table 4, Figure 4 and Figure S4). In the control foam Ref-0%P, the maximum force *F_max_* of approximately 270 kPa was reached at low compression. The addition of TEP in TEP-0.3%P resulted in an increase of *F_max_* to about 350 kPa. The foam TPP-0.7%P without TEP, but with TPP showed, as expected, a lower maximum force that was comparable to Ref-0%P. Substitution of TPP by the BPPO-containing FR additives induced slight changes to the compression strength as compared to TPP/TEP-1.0%P with EA-BPPO/TEP-1.0%P having *F_max_* at slightly higher value and MA-BPPO/TEP-1.0%P having *F_max_* at a slightly lower value. It seems to be surprising that a plasticizer, like TEP, led to higher compression strength. This effect can be explained by the reduction of the glass transition temperature *T_g_* by TEP with reaction time (*T_g_* = *f*(*t_reaction_*)). The improvement in mobility of polymer chains during the polymerization led to increased levels of diffusion-controlled trimerization. Higher degrees of trimerization induce higher crosslinking densities, and this is visible in the compression strength. 

### 3.3. Thermal Decomposition of the Foams

**Figure 5:**
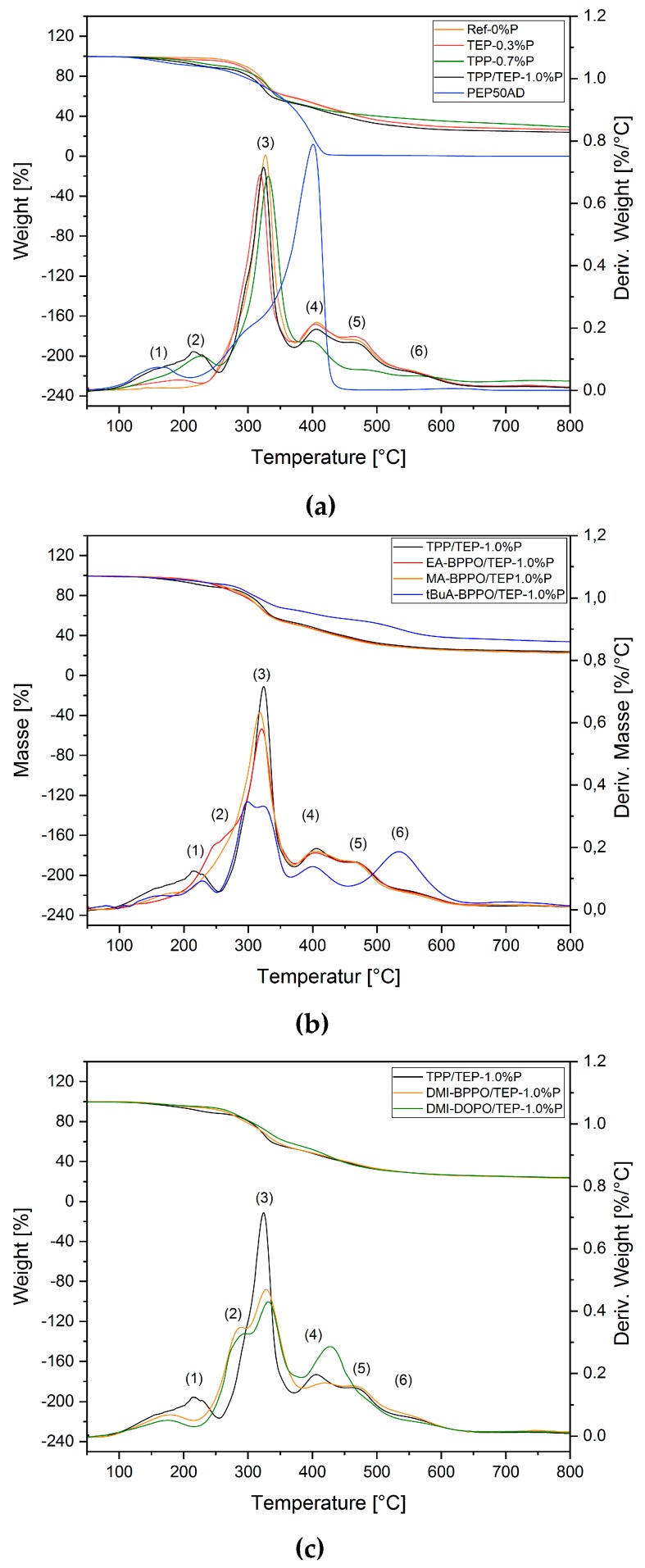
Thermogravimetric analysis (TGA) results: (**a**) TGA curves of benchmark foams and polyol used; (**b**) TGA curves of the benchmark foam compared to foams containing BPPO-derivatives; and, (**c**) TGA curves of the benchmark foam compared with a foam with BPPO-derivative and a DOPO-derivative. All the formulations shown here are specified in Table S1 in the Supporting Information.

The thermal decomposition of the foams was investigated by TGA under nitrogen up to 800 °C, in combination with pyrolysis-GC/MS executed at the decomposition maxima found in TGA. Table 5 summarizes the results together with a comparison of the residues that are found at the end of the cone calorimeter test (Section 2.4.2.). The pyrolysis products that evolved in the gas phase and were observed by pyrolysis-GC/MS are summarized in Table S4 in the Supplementary Materials. The TGA curves in Figure 5 illustrate the decomposition.

The addition of TEP in TEP-0.3%P and TPP/TEP-1.0%P (Figure 5a) did not alter the main decomposition maximum (3) in the temperature range of 320 °C, but induced a new maximum (1) at a lower temperature (192 °C). This maximum is connected to the vaporization of TEP, as reported previously [2] and proven by pyrolysis-GC/MS (Supplementary Materials, Table S4). Consequently, it was found in all samples containing TEP.

The addition of TPP caused a new maximum (2) at 216 °C that is connected to the evaporation of TPP [39,40,41], which was proven by pyrolysis-GC/MS. The flame-retardant effect of both TEP and TPP originates from their vaporization at temperatures that are lower than *T_max_* (3). The residues of Ref-0%P, TEP-0.3%P, and TPP/TEP-1.0%P found at 800 °C were comparable. These observations suggest that both TPP and TEP completely evaporate in the gas phase.

Figure 5b illustrates the thermal decomposition of foams, where the acrylate-BPPO additives substituted TPP. When EA-BPPO was used instead of TPP, a slight change of the TGA curve was visible; the shift of maximum (2) to higher temperature (248 °C) was attributed to the vaporization of EA-BPPO or BPPO fragments. The structurally comparable MA-BPPO caused further changes. The shift of maximum (2) towards maximum (3) and of maximum (3) to a lower temperature was observed, as well as the disappearance of maximum (5). tBuA-BPPO led to the occurrence of a double peak. The structure of the t-butyl group additionally resulted in a strong enhancement of maximum (6) and induced charring. This correlated to a significantly higher residue at 800 °C (char increased by 10 wt.% as compared to TPP/TEP-1.0%P).

The pyr-GC/MS spectra of all the samples taken around 300 °C, i.e., at decomposition maximum (3) indicated the formation of diethylene glycol and phthalic acid anhydride or phthalic acid, respectively. These products are expected for the used polyester polyol co-monomer (see chemical structure of the polyester polyol in Figure 2). All other decomposition compounds were found in minor concentrations. Table S4 summarizes all of the decomposition products that were found in the samples studied for the respective temperature; Figures S5–S10 show the mass spectra that were obtained.

### 3.4. Fire Behavior of the Foams

#### 3.4.1. Vertical Flame Spread

Table 6 summarizes the flame length observed in the VFS test. The control foam Ref-0%P without phosphorus (TEP or TPP) completely burned. The addition of TEP in TEP-0.3%P led to a slight reduction of the flame length. Adding the benchmark FR TPP, the foam TPP/TEP-1.0%P could have passed the “B2” qualification with a VFS of 15 cm.

Foams with nearly all BPPO-acrylates and itaconates passed the VFS-specification of DIN 4102 at a phosphorus concentration of 1 wt.%. Increasing the P-concentration further reduced the flame spread. A comparison of BPPO-itaconate containing foams with DMI-DOPO containing foams showed that the latter had a lower efficiency. Formulations with higher P-contents could not be properly homogenized and they yielded heterogeneous foams. The use of the hydroquinone additive HQ-BPPO led to foams that did not pass the VFS specification, while HQ-DOPO led to a very low flame spread. The foams that were prepared with the amide AM-BPPO yielded flame lengths higher than 15 cm, thus not passing the VFS specification. Even if the P-content was increased, the test could not be passed with AM-BPPO. One of the reasons might be the different density of these foams. A second explanation could be that the hydroquinones and amides are not inert as solids, but rather react with isocyanate to form urethane and acylurea, leading to lower NCO/OH ratios than the other foams—although NMR could not prove this reaction. It is known that both aromatic urethanes and acylureas are unstable beyond 120 °C, adding further complexity to the polymerization reaction.

The VFS test yields results that give an indication of FR activity. More detailed results are provided by cone calorimetry under forced flaming conditions, as discussed in the next section.

#### 3.4.2. Forced Flaming Combustion

Foams with EA-BPPO, MA-BPPO, tBuA-BPPO, DMI-BPPO, and DMI-DOPO having a roughly similar density and pore sizes were selected for the examination of the developing fire behavior in forced flaming combustion by cone calorimeter. HQ-BPPO and HQ-DOPO were analyzed, despite the different densities to expand the comparison between BPPO and DOPO additives.

**Figure 6:**
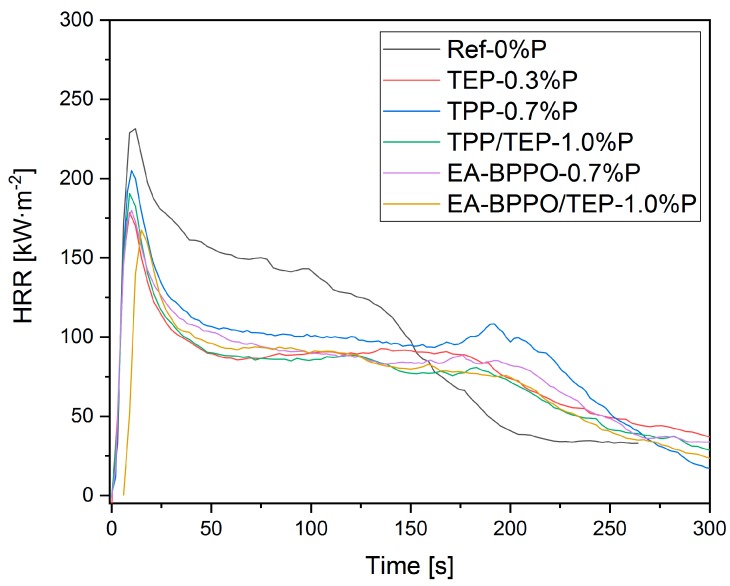
Heat release rate curves (average of three measurements) obtained by forced flaming combustion with heat flux 50 kW·m^−2^ of control foams (Ref-0%P, TEP-0.3%P, TPP-0.7%P, and TPP/TEP-1.0%P) as compared to the foams EA-BPPO-0.7%P and EA-BPPO/TEP-1.0%P.

Figure 6 compares the HRR over time of foam Ref-0%P to foams with EA-BPPO, TEP, and TPP. The curve for the control foam Ref-0%P is roughly comparable with that results that were reported in the literature [3], although the pentane levels in the cells here and in the literature differ significantly. The foam TEP-0.3%P showed a reduction of HRR in the steady burning phase between 50 and 275 s. The addition of TPP in TPP/TEP-1.0%P did not alter the first phase of burning until 200 s, but it reduced the HRR after this time. When TPP was substituted by EA-BPPO in EA-BPPO/TEP-1.0%P the pentane-related first peak appeared later, as desired, and it was lower than for the benchmark foam TPP/TEP-1.0%P.

Table 7 summarizes the results of the measurements (selected parameters). The complete data set is given in the SI (Table S6).

**Figure 7:**
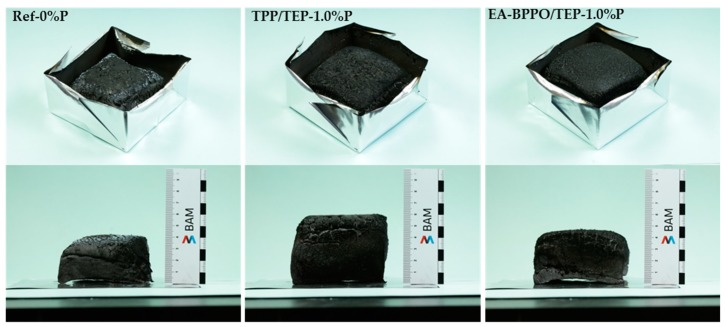
Images of foam Ref-0%P, TPP/TEP-1.0%P, and EA-BPPO/TEP-1.0%P after the cone calorimeter test.

All of the foams ignited after 1 s. This is typical for insulating foams with low density that do not dissipate the heat from the heater into the material [42]. The maximum of the heat release rate (PHRR) was reached 10 s after ignition in all of the samples. Fast flame spread after ignition is followed by fast formation of a charring layer, which lowers the heat release. For most foams, only little shrinkage was observed which can be attributed to the crosslinking density of the PIR foams. Figure 7 illustrates the appearance of selected foams after the cone calorimeter test.

The control foam Ref-0%P left substantial residue with a height of 4 cm after the cone experiment. This illustrates the efficiency of the isocyanurate rings in char formation. The addition of TEP alone in TEP-0.3%P raised the char height to 4.5 cm, and the further addition of condensed phase-active TPP to 6 cm. Exchange of TPP by EA-BPPO in EA-BPPO/TEP-1.0%P altered the char height to 4.5 cm, a height that was observed in all foams with BPPO additives. Therefore, the chars of foams with BPPO additives had higher density. The phosphorus additives enhanced the residue to about 30 wt.% in each case. Furthermore, increasing the P-content raised the residue accordingly.

**Figure 8:**
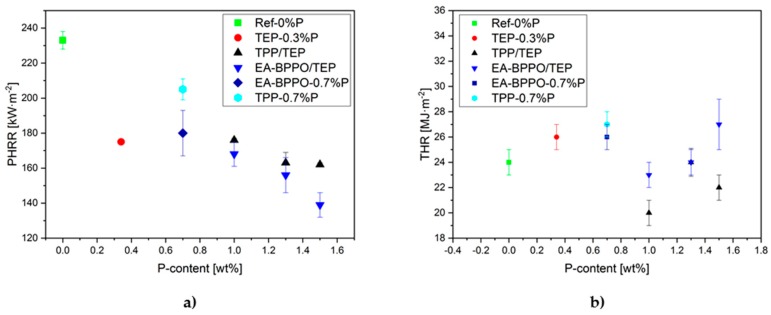
Correlation of (**a**) peak heat release rate (PHRR) and (**b**) total heat released (THR) of selected foams to their P-content.

The addition of TEP resulted in a drastic decrease in PHRR (TEP-0.3%P). Further addition of TPP in TPP/TEP-1.0%P formulation did not alter the PHRR. The same applies for MARHE. The substitution of TPP by the BPPO derivatives to meet the comparable P-content (1 wt.%) yielded PHRR and MARHE values that were comparable to TPP/TEP-1.0%P. The PHRR values that were found correlated to the P-content, except for MA-BPPO/TEP-1.4%P, Figure 8. THR was hardly influenced by all the additives that we ascribe to the comparable P-contents and the comparable amount of burnt material. The effective heat of combustion was also hardly affected by the type of FR additives.

The dimethyl itaconates were used to compare DOPO and BPPO at a phosphorus loading of 1 wt.%. Significant differences between DMI-DOPO (DMI-DOPO/TEP-1.0%P) and DMI-BPPO (DMI-BPPO/TEP-1.0%P) were not found in the cone calorimeter tests. Both performed in the range of TPP/TEP-1.0%P benchmark, except for a lower TSR (Table 7). The TSR of both samples was lower than that with the BPPO-acrylates. The CO yield in the samples with BPPO-based FR, except for the itaconate, was reduced by 10% when compared to the benchmark TPP/TEP-1.0%P (Supplementary Materials, Table S6). The sample HQ-DOPO/TEP-1.0%P showed a clear but reproducible irregularity in the parameters (higher PHRR, MARHE, TSR), which we attribute to the higher density of the foam. Higher density resulted from the altered reaction kinetics (see Section 3.2.).

**Figure 9:**
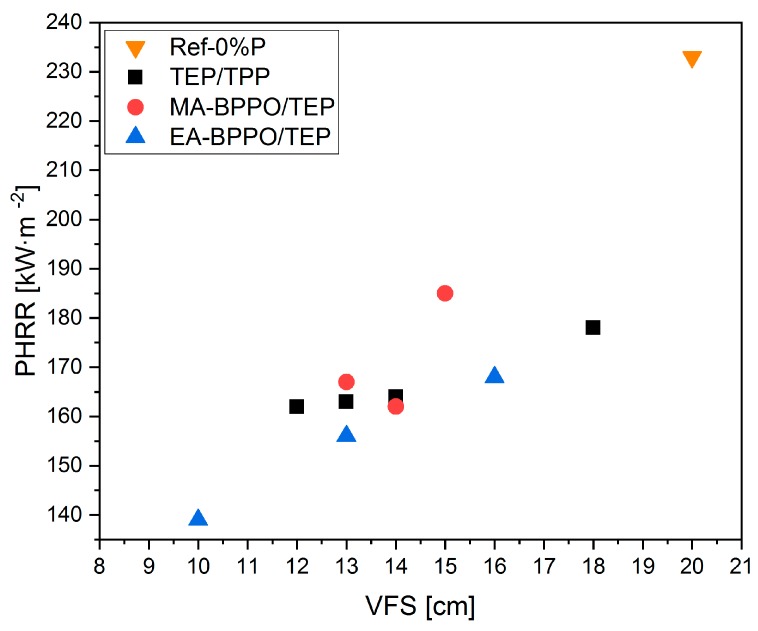
Correlation between PHRR and VFS of the reference foams, MA-BPPO/TEP, and EA-BPPO/TEP.

Figure 9 compares vertical flame spread and the maximum heat release rate. They both follow the same trend: samples with lower PHRR (i.e., with higher P-content) also show lower VFS.

## 4. Conclusions

A series of new flame retardants (FR) based on dibenzo[d,f][1,3,2]dioxaphosphepine 6-oxide (BPPO) incorporating acrylates and benzoquinone were tested in PUR/PIR foams with respect to their fire performance. All FR agents were tested at comparable P-concentrations. The majority of the novel BPPO derivatives did not alter the physical properties (density and morphology) of the PIR foams. We found differences in the FR properties (PHRR, MARHE and TSR) based on the substituents of the acrylate employed. At 1 wt% P, the structurally more complex tBuA-BPPO turned out to be the most effective FR additive due to lower PHRR, MARHE, TSR as well as a higher residue compared to the benchmark triphenyl phosphate (phosphorus oxidation state +V). The majority of the BPPO-acrylate and itaconate derivatives behaved similar to the the benchmark, triphenyl phosphate. The novel FR were also compared to chemically similar substances based on 9,10-dihydro-9-oxy-10-phosphaphenanthrene-10-oxide (DOPO) with phosphorus in the oxidation state –I to investigate the influence of oxidation state on gas phase and condensed phase contributions to flame retardancy in PUR/PIR foams. In forced flaming combustion experiments a significant influence of the FR on charring, TSR, and MARHE was found. An enhanced charring behavior for the P(+III) compounds compared to P(-I) could be confirmed according to statements in literature **[2]**. This agrees with the higher gas phase activity of P(+I) derivatives observed. In summary, the BPPO-containing foams combine fire protection with increased compression strength. These findings have practical relevance for tuning FR properties of PIR foams to meet safety and regulatory requirements, e.g., in the building and construction industry.

## Figures and Tables

**Table 1:** BPPO derivatives studied as flame retardant (FR) additives in polyurethane foams.

Entry	BPPO Added to…	R_1_	Chemical Structure	Abbreviation
**1**	Methyl acrylate	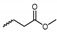	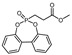	MA-BPPO
**2**	Ethyl acrylate	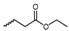	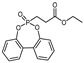	EA-BPPO
**3**	Tert. Butyl acrylate	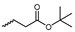	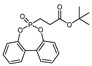	tBuA-BPPO
**4**	Acrylamide	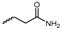	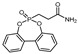	AM-BPPO
**5**	Phenyl acrylate	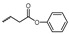	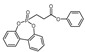	PA-BPPO
**6**	Dimethyl fumarate	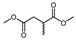	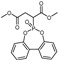	SU-BPPO
**7**	Dimethyl itaconate	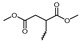	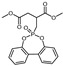	DMI-BPPO
**8**	Diphenyl fumarate	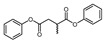	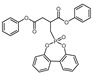	DPF-BPPO
**9**	p-Benzoquinone	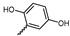	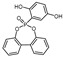	HQ-BPPO
**10**	p-Benzoquinone	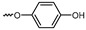	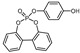	HP-BPPO
	**DOPO added to…**			
**11**	Dimethyl itaconate	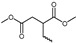	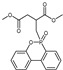	DMI-DOPO
**12**	p-Benzoquinone	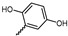	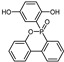	HQ-DOPO

**Table 2:** Formulation of the polyisocyanurate (PIR) foams studied.

Ingredient	Amount
	[g]
Stabilizer (TEGOSTAB B 8421)	4.0
Emulsifier (Emulsogen TS100)	2.0
Triethyl phosphate (TEP)	5.0
PEG 400	16.0
Catalyst (KAc 25 wt.% in diethylene glycol)	2.5
Polyester polyol (PEP50 AD)	53.0
Blowing Agent (pentane)	15.0
Flame Retardant ^a^	x
Polyisocyanate (DESMODUR 44V70L)	151.8

^a^ Amount depends on the P-content of the FR according to Table 1 itself and the P-content desired (1 wt.%) in the foam.

**Table 3:** Physical properties of the studied foams (description of methods: see Experimental part).

Foam	P-content	Density	*WA_V _*	Pore size	Cream Time	Setting Time	Rising Time
	[wt.%]	[kg·m^−^^3^]	[vol%]	[mm]	[s]	[s]	[s]
**Ref-0%P**	0.0	36	7	0.15	12	30	41
**TEP-0.3%P**	0.3	36	4	0.18	10	23	31
**TPP-0.7%P**	0.7	36	7	0.17	16	30	40
**TPP/TEP-1.0%P**	1.0	39	5	0.15	10	24	32
**TPP/TEP-1.3%P**	1.3	40	10	0.14	14	25	35
**TPP/TEP-1.5%P**	1.5	39	12	0.10	13	23	30
**MA-BPPO/TEP-1.0%P**	1.0	38	4	0.17	10	35	45
**MA-BPPO/TEP-1.2%P**	1.2	39	14	0.15	14	38	48
**MA-BPPO/TEP-1.4%P**	1.4	37	9	0.17	13	48	58
**EA-BPPO-0.7%P**	0.7	36	7	0.29	16	40	45
**EA-BPPO/TEP-1.0%P**	1.0	37	6	0.19	9	26	33
**EA-BPPO/TEP-1.3%P**	1.3	39	6	0.21	7	27	37
**EA-BPPO/TEP-1.4%P**	1.4	42	20	0.16	12	38	47
**EA-BPPO/TEP-1.5%P**	1.5	42	3	0.20	15	28	35
**tBuA-BPPO/TEP-1.0%P**	1.0	39	19	0.14	11	31	41
**AM-BPPO/TEP-1.0%P**	1.0	51	13	0.18	11	33	40
**AM-BPPO/TEP-1.3%P**	1.3	50	12	0.12	13	37	45
**AM-BPPO/TEP-1.5%P**	1.5	57	3	0.17	20	50	50
**PA-BPPO/TEP-1.0%P**	1.0	39	16	0.18	12	32	40
**SU-BPPO/TEP-1.0%P**	1.0	50	23	0.23	12	70	42
**DPF-BPPO/TEP-1.0%P ^a^**	1.0	-	-	-	15	>200	51
**DMI-BPPO/TEP-1.0%P**	1.0	35	16	0.16	11	37	46
**DMI-BPPO/TEP-1.5%P**	1.5	47	13	0.15	10	30	41
**HQ-BPPO/TEP-1.0%P**	1.0	55	8	0.19	10	15	15
**HQ-BPPO/TEP-1.5%P**	1.5	58	22	0.33	5	15	15
**HP-BPPO/TEP-1.0%P**	1.0	39	14	0.19	6	22	29
**DMI-DOPO/TEP-1.0%P**	1.0	35	11	0.14	12	31	40
**DMI-DOPO/TEP-1.5%P**	1.5	42	19	0.20	13	44	50
**HQ-DOPO/TEP-1.0%P**	1.0	39	2	0.19	8	23	30
**HQ-DOPO/TEP-1.5%P**	1.5	45	23	0.19	5	16	16

^a^ Yielded unstable foam.

**Table 4:** Mechanical properties of selected foams.

Foam	P-content	*F_max_*	*F*_60%_^a^
	[wt.%]	[kPa]	[kPa]
**Ref-0%P**	0	271	197
**TEP-0.3%P**	0.3	347	255
**TPP/TEP-1.0%P**	1.0	353	261
**TPP-0.7%P**	0.7	282	184
**EA-BPPO/TEP-1.0%P**	1.0	363	234
**MA-BPPO/TEP-1.0%P**	1.0	314	245

^a^ Value before unloading at ε = 60%.

**Table 5:** Decomposition characteristics of the studied foams (description of methods: see Experimental part).

Foam	P-Content	TGA *T_max_*	TGA Residue	Fire Residue
	[wt.%]	[°C]	[wt.%]	[wt.%]
**Ref-0%P**	0.0	326	26.6	22.3
**TEP-0.3%P**	0.3	319	26.4	26.7
**TPP-0.7%P**	0.7	330	29.3	44.7
**TPP/TEP-1.0%P**	1.0	324	24.0	36.4
**TPP/TEP-1.3%P**	1.3	328	23.8	27.8
**TPP/TEP-1.5%P**	1.5	331	19.6	28.6
**MA-BPPO/TEP-1.0%P**	1.0	318	22.9	28.3
**MA-BPPO/TEP-1.2%P**	1.2	320	24.6	33.1
**MA-BPPO/TEP-1.4%P**	1.4	315	25.0	34.1
**EA-BPPO-0.7%P**	0.7	331	24.6	28.2
**EA-BPPO/TEP-1.0%P**	1.0	321	22.6	28.6
**EA-BPPO/TEP-1.3%P**	1.3	314	21.3	29.2
**EA-BPPO/TEP-1.4%P**	1.4	329	22.0	-
**EA-BPPO/TEP-1.5%P**	1.5	319	23.7	35.7
**tBuA-BPPO/TEP-1.0%P**	1.0	308	33.4	27.0
**AM-BPPO/TEP-1.0%P**	1.0	316	30.0	-
**AM-BPPO/TEP-1.3%P**	1.3	295	31.2	-
**AM-BPPO/TEP-1.5%P**	1.5	319	30.3	-
**PA-BPPO/TEP-1.0%P**	1.0	320	34.6	-
**SU-BPPO/TEP-1.0%P**	1.0	334	28.5	-
**DMI-BPPO/TEP-1.0%P**	1.0	334	29.1	33.2
**DMI-BPPO/TEP-1.5%P**	1.5	319	23.6	-
**HQ-BPPO/TEP-1.0%P**	1.0	334	33.5	31.7
**HQ-BPPO/TEP-1.5%P**	1.5	315	28.9	-
**HP-BPPO/TEP-1.0%P**	1.0	321	28.1	-
**DMI-DOPO/TEP-1.0%P**	1.0	331	23.9	29.4
**DMI-DOPO/TEP-1.5%P**	1.5	278	24.5	-
**HQ-DOPO/TEP-1.0%P**	1.0	325	23.3	38.0
**HQ-DOPO/TEP-1.5%P**	1.5	327	23.8	-

**Table 6:** Results of vertical flame spread vertical flame spread (VFS) test (DIN 4102).

Foam	P-content	Density	VFS
	[wt.%]	[kg·m^−3^]	[cm]
**Ref-0%P**	0.0	36	>20
**TEP-0.3%P**	0.3	36	18
**TPP-0.7%P**	0.7	36	15
**TPP/TEP-1.0%P**	1.0	39	14
**TPP/TEP-1.3%P**	1.3	40	13
**TPP/TEP-1.5%P**	1.5	39	12
**MA-BPPO/TEP-1.0%P**	1.0	35	15
**MA-BPPO/TEP-1.2%P**	1.2	47	14
**MA-BPPO/TEP-1.4%P**	1.4	35	13
**EA-BPPO-0.7%P**	0.7	36	17
**EA-BPPO/TEP-1.0%P**	1.0	42	16
**EA-BPPO/TEP-1.3%P**	1.3	38	13
**EA-BPPO/TEP-1.4%P**	1.4	39	13
**EA-BPPO/TEP-1.5%P**	1.5	37	<10
**tBuA-BPPO/TEP-1.0%P**	1.0	37	15
**AM-BPPO/TEP-1.0%P**	1.0	51	18
**AM-BPPO/TEP-1.3%P**	1.3	50	18
**AM-BPPO/TEP-1.5%P**	1.5	57	18
**PA-BPPO/TEP-1.0%P**	1.0	39	11
**SU-BPPO/TEP-1.0%P**	1.0	50	18
**DMI-BPPO/TEP-1.0%P**	1.0	39	15
**DMI-BPPO/TEP-1.5%P**	1.5	42	<10
**HQ-BPPO/TEP-1.0%P**	1.0	55	18
**HQ-BPPO/TEP-1.5%P**	1.5	58	18
**HP-BPPO/TEP-1.0%P**	1.0	39	17
**DMI-DOPO/TEP-1.0%P**	1.0	42	14
**DMI-DOPO/TEP-1.5%P**	1.5	39	16
**HQ-DOPO/TEP-1.0%P**	1.0	39	<10
**HQ-DOPO/TEP-1.5%P**	1.5	45	<10

**Table 7:** Results of cone calorimeter tests on the PIR foams under study (heat flux 50 kW·m^−2^).

Foam	PHRR	MARHE	THR	Residue	EHC	TSR
	[kW·m^−2^]	[kW·m^−2^]	[MJ·m^−2^]	[wt.%]	[MJ·kg^−1^]	[m^2^·m^−2^]
**Ref-0%P**	233	172	24	22.3	2.0	551
**TEP-0.3%P**	178	128	26	26.7	2.1	392
**TPP-0.7%P**	205	145	27	44.7	2.1	755
**TPP/TEP-1.0%P**	164	111	20	36.4	1.8	324
**TPP/TEP-1.3%P**	163	114	24	27.8	1.8	407
**TPP/TEP-1.5%P**	162	112	22	28.6	1.8	389
**MA-BPPO/TEP-1.0%P**	185	132	28	28.3	2.2	527
**MA-BPPO/TEP-1.2%P**	162	112	23	33.1	2.0	421
**MA-BPPO/TEP-1.4%P**	167	114	25	34.4	1.9	488
**EA-BPPO-0.7%P**	180	132	26	28.2	2.0	501
**EA-BPPO/TEP-1.0%P**	168	121	23	28.6	1.9	433
**EA-BPPO/TEP-1.3%P**	156	113	24	29.2	1.9	506
**EA-BPPO/TEP-1.5%P**	139	103	27	35.7	2.1	398
**tBuA-BPPO/TEP-1.0%P**	166	114	26	27.0	2.0	380
**DMI-BPPO/TEP-1.0%P**	171	118	21	33.2	1.9	317
**DMI-DOPO/TEP-1.0%P**	162	108	24	29.4	1.9	371
**HQ-BPPO/TEP-1.0%P ^a^**	374	266	29	31.7	1.7	1228
**HQ-DOPO/TEP-1.0%P**	134	101	22	38.0	1.8	503

^a^ Deviating density (50 kg·m^−3^).

## Supplementary Materials

The following supplementary materials are available online at https://www.mdpi.com/2073-4360/11/8/1242/s1, Figure S1. ATR-FTIR spectra of selected foams, Figure S2. FTIR spectra of pure EA-BPPO, Figure S3. ATR-FTIR spectra of foam Ref-0%P and EA-BPPO/TEP-1.0%P compared with pure EA-BPPO, Figure S4. Stress-strain diagrams of the foams selected foams, Figure S5. Pyr-GC/MS spectra of Ref-0%P, Figure S6. Pyr-GC/MS spectra of TEP-0.3%P, Figure S7. Pyr-GC/MS spectra of TPP/TEP-1.0%P, Figure S8. Pyr-GC/MS spectra of EA-BPPO/TEP-1.0%P, Figure S9. Pyr-GC/MS spectra of DMI-BPPO/TEP-1.0%P, Figure S10. Pyr-GC/MS spectra of DMI-DOPO/TEP-1.0%P, Table S1. Foam compositions studied, Table S2. Polycondensation experiments of SU-BPPO with 1,4-butanediol, Table S3. Transesterification experiments with EA-BPPO and 1,4-butanediol, Table S4. Decomposition products observed with Pyr-GC/MS, Table S5. Quantitative P-contents of selected foams and their residues after cone calorimetry, Table S6. Complete results of cone calorimeter tests on the PIR foams under study.
