# Supplementary material for: Improving the Flame Retardance of Polyisocyanurate Foams by Dibenzo[d,f][1,3,2]dioxaphosphepine 6-Oxide-Containing Additives
Source: Polymers (Basel). 2019 Jul 26;11(8):1242. doi: 10.3390/polym11081242 (PMC6723653; doi:10.3390/polym11081242)
Supplement: Supplementary file 1 [file polymers-11-01242-s001.pdf]

Article

BPPO as a phosphonate analogue of DOPO and the use of its derivatives as flame retardants in polyisocyanurate foams

Johannes Lenz[1,2], Doris Pospiech[1], Maxime Paven[3], Rolf Albach[3], Martin Günther[4], Bernhard Schartel[4], Brigitte Voit[1,2]*

[1] Leibniz-Institut für Polymerforschung Dresden e.V., Germany
[2] Technische Universität Dresden, Organic Chemistry of Polymers, Germany
[3] Covestro Deutschland AG, 51365 Leverkusen, Germany
[4] Bundesanstalt für Materialforschung und -prüfung (BAM), 12205 Berlin, Germany
*** Correspondence: pospiech@ipfdd.de; Tel.: +49 351 4658 497**

Supporting Information

Outline

1. Foam compositions studied 2
2. ATR- FTIR spectra 3
3. Polycondensation / transesterification experiments 4
4. Mechanical properties of the foams 5
5. Pyr-GC-MS 6
6. Quantitative determination of phosphorus contents 11
7. Cone calorimetry 12

1. Foam compositions studied

Table S1. Foam compositions studied.

Foam	TEGOSTAB B8421	Emulsogen TS100	TEP	PEG 400	Cat.alyst*	PEP50AD	Pentane	44V70L	FR	Mass of FR [g]	P [wt%] in foam
**Ref-0%P**	4	2	-	16	2.5	53	15	151.8	-	0	0.0
**TEP-0.7%P**	4	2	5	16	2.5	53	15	151.8	Only TEP	0	0.3
**TPP/TEP-1.0%P**	4	2	5	16	2.5	53	15	151.8	TPP	19	1.0
**TPP-0.7%P**	4	2	-	16	2.5	53	15	151.8	TPP, no TEP	19	0.7
**EA-BPPO-0.7%P**	4	2	-	16	2.5	53	15	158.8	EA-BPPO	19	0.7
**EA-BPPO/TEP-1.0%P**	4	2	5	16	2.5	53	15	151.8	EA-BPPO	20	1.0
**EA-BPPO/TEP-1.3%P**	4	2	5	16	2.5	53	15	151.8	EA-BPPO	30	1.3
**EA-BPPO/TEP-1.4%P**	4	2	5	16	2.5	53	15	151.8	EA-BPPO	35	1.4
**EA-BPPO/TEP-1.5%P**	4	2	5	16	2.5	53	15	151.8	EA-BPPO	37	1.5
**MA-BPPO/TEP-1.0%P**	4	2	5	16	2.5	53	15	151.8	MA-BPPO	19	1.0
**MA-BPPO/TEP-1.2%P**	4	2	5	16	2.5	53	15	151.8	MA-BPPO	26	1.2
**MA-BPPO/TEP-1.4%P**	4	2	5	16	2.5	53	15	151.8	MA-BPPO	33	1.4
**tBuA-BPPO/TEP-1.0%P**	4	2	5	16	2.5	53	15	151.8	tBuA-BPPO	22	1.0
**PA-BPPO/TEP-1.0%P**	4	2	5	16	2.5	53	15	151.8	PA-BPPO	23	1.0
**AM-BPPO/TEP-1.0%P**	4	2	5	16	2.5	53	15	151.8	AM-BPPO	18	1.0
**AM-BPPO/TEP-1.3%P**	4	2	5	16	2.5	53	15	151.8	AM-BPPO	27	1.3
**AM-BPPO/TEP-1.5%P**	4	2	5	16	2.5	53	15	151.8	AM-BPPO	33	1.5
**SU-BPPO/TEP-1.0%P**	4	2	5	16	2.5	53	15	151.8	SU-BPPO	24	1.0
**DPF-BPPO/TEP-1.0%P**	4	2	5	16	2.5	53	15	151.8	DPF-BPPO	32	1.0
**DMI-BPPO/TEP-1.0%P**	4	2	5	16	2.5	53	15	151.8	DMI-BPPO	25	1.0
**DMI-BPPO/TEP-1.5%P**	4	2	5	16	2.5	53	15	151.8	DMI-BPPO	45	1.5
**HP-BPPO/TEP-1.0%P**	4	2	5	16	2.5	53	15	151.8	HP-BPPO	20	1.0
**HQ-DOPO/TEP-1.0%P**	4	2	5	16	1.5	53	15	151.8	HQ-DOPO	17	1.0
**DMI-DOPO/TEP-1.5%P**	4	2	5	16	2.5	53	15	151.8	DMI-DOPO	42.5	1.5
**DMI-DOPO/TEP-1.0%P**	4	2	5	16	2.5	53	15	151.8	DMI-DOPO	22	1.0

2. ATR- FTIR spectra

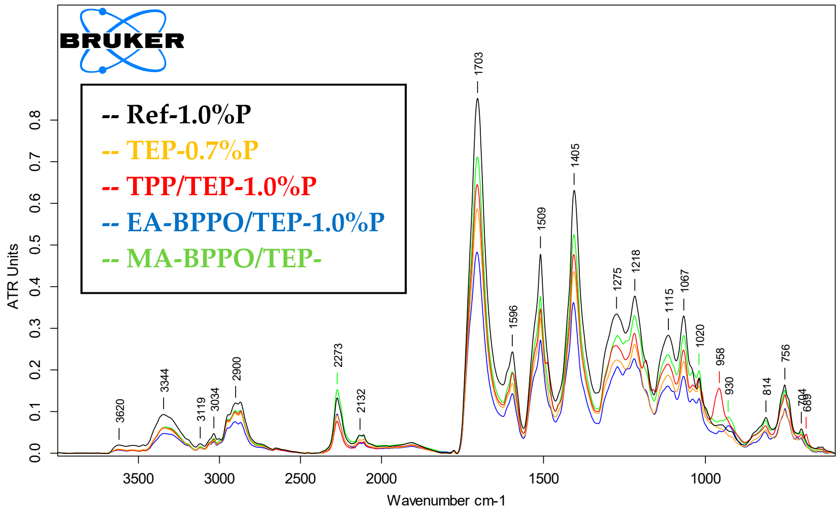

Figure S1. ATR-FTIR spectra of the selected foams.

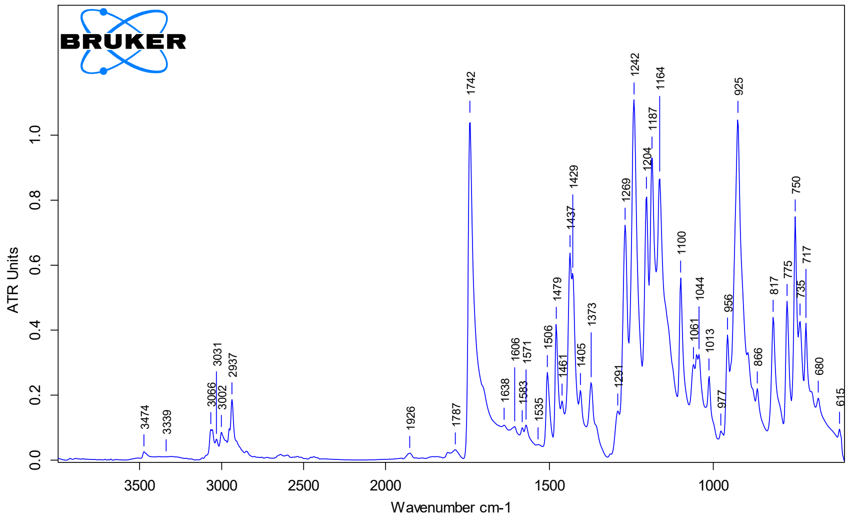

Figure S2. FTIR spectra of pure EA-BPPO.

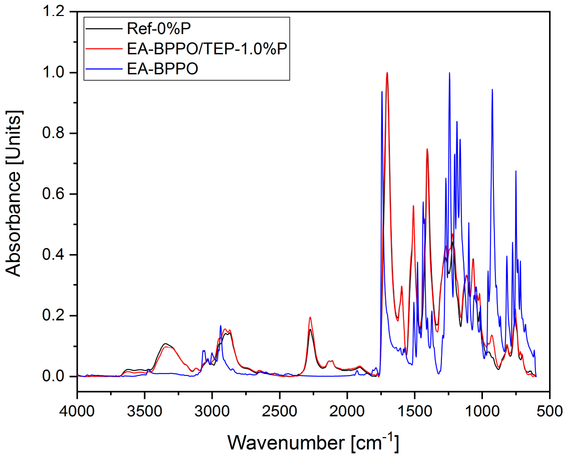

Figure S3. ATR-FTIR spectra of foam **Ref-0%P** and **EA-BPPO/TEP-1.0%P** compared with pure EA-BPPO.

3. Polycondensation / transesterification experiments

Table S2. Polycondensation experiments of SU-BPPO with 1,4-butanediol.

Catalyst	cat* [wt%]	Molar excess**	T [°C]	t [h]	vacuum [min]
$Ti(OBu)_4$+ Sb_2O_3	0.5 + 0,5	3	150-230	1.5	30
$Ti(OBu)_4$	0.5	2	150-230	1.5	30
$Ti(OBu)_4$	0.5	2	100	72	-
$Ti(OBu)_4$	0.5	2	80	72	-
KAc	0.5	2	100	72	-
KAc	0.5	2	100	24	-
$Ti(OBu)_4$	0.5	3	100	24	-
$Ti(OBu)_4$	1.0	2	100	24	-
LiAc	1.0	3	100	24	-
$Ti(OBu)_4$	1.0	2	100	72	30
$Ti(OBu)_4$	1.5	2	100	24	-
$Ti(OBu)_4$	1.0	2	100	72	30
$Ti(OBu)_4$ + Sb_2O_3	0.5 + 0.5	2	100	72	30
$Ti(OBu)_4$	1.0	2	120	4	30
$Ti(OBu)_4$	1.0	2	100	4	30
$SnCl_2$ x 2 H_2O	1.0	2	100	72	30
Dibutyltinndilaurate	1.0	2	100	72	30
$Ti(OBu)_4$	1.0	2	120	24	30
$Ti(OBu)_4$	1.0	5	150	4	30
Tin(II) 2-ethylhexanoate	2.0	2	150	24	30

* with respect to to diester
** excess of 1,4-butanediole

Table S3. Transesterification experiments with EA-BPPO and 1,4-butanediol.

Catalyst	cat* [wt%]	Molar excess**	T [°C]	t [h]	vacuum [min]
$Ti(OBu)_4$	1	3	150-230	1.5	30
$Ti(OBu)_4$	1	1	150	20	30
$Ti(OBu)_4$	1	1 (Ethanediol)	150	4	30
$Ti(OBu)_4$	1	5	100	72	-
$Ti(OBu)_4$	1	3	150	4	4
$Ti(OBu)_4$	1	5	150-200	1.5	30

* with respect to diester
** excess of butanediole

4. Mechanical properties of the foams

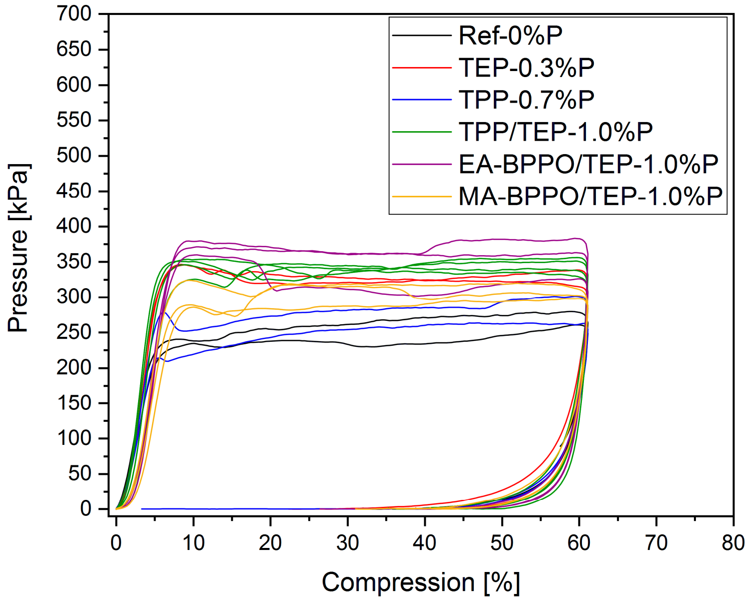

Figure S4. Stress-strain diagrams of selected foams.

5. Pyr-GC-MS

Table S4. Decomposition products observed with Pyr-GC-MS.

Foam	T [°C]	Diethylene glycol	2-(2-chloroethoxy)ethan-1-ol	Benzaldehyde	Aniline	Dimethyl itaconate	Dodecanylchloride	TEP	Phthalic acid anhydride	Aminotoluene	Phthalic acid	Benzylaniline	MDI	Dibutylterephthalate	TPP
**Ref-0%P**	327	+									+		+		
	468	+			+							+			
**TEP-0.3%P**	190							+							
	319	+									+				
	405	+									+				
	465				+					+		+			
**TPP/TEP-1.0%P**	215			+				+							
	324	+									+				+
	465	+			+						+				
**EA-BPPO/TEP-1.0%P**	130							+							
	245							+							
	465	+							+				+		
**DMI-BPPO/TEP-1.0%P**	167					+		+							
	284	+					+		+						
	330	+							+						
**DMI-DOPO/TEP-1.0%P**	174							+						+	
	250	+	+			+			+						
	425		+						+						

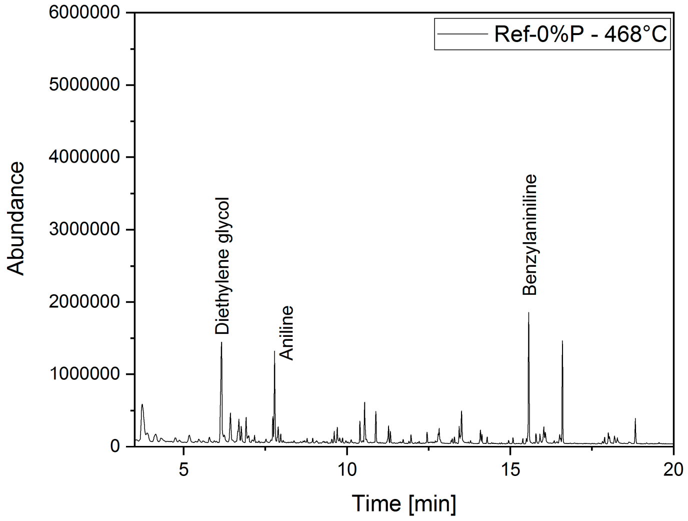

Figure S5. Pyr-GC/MS spectra of **Ref-0%P.**

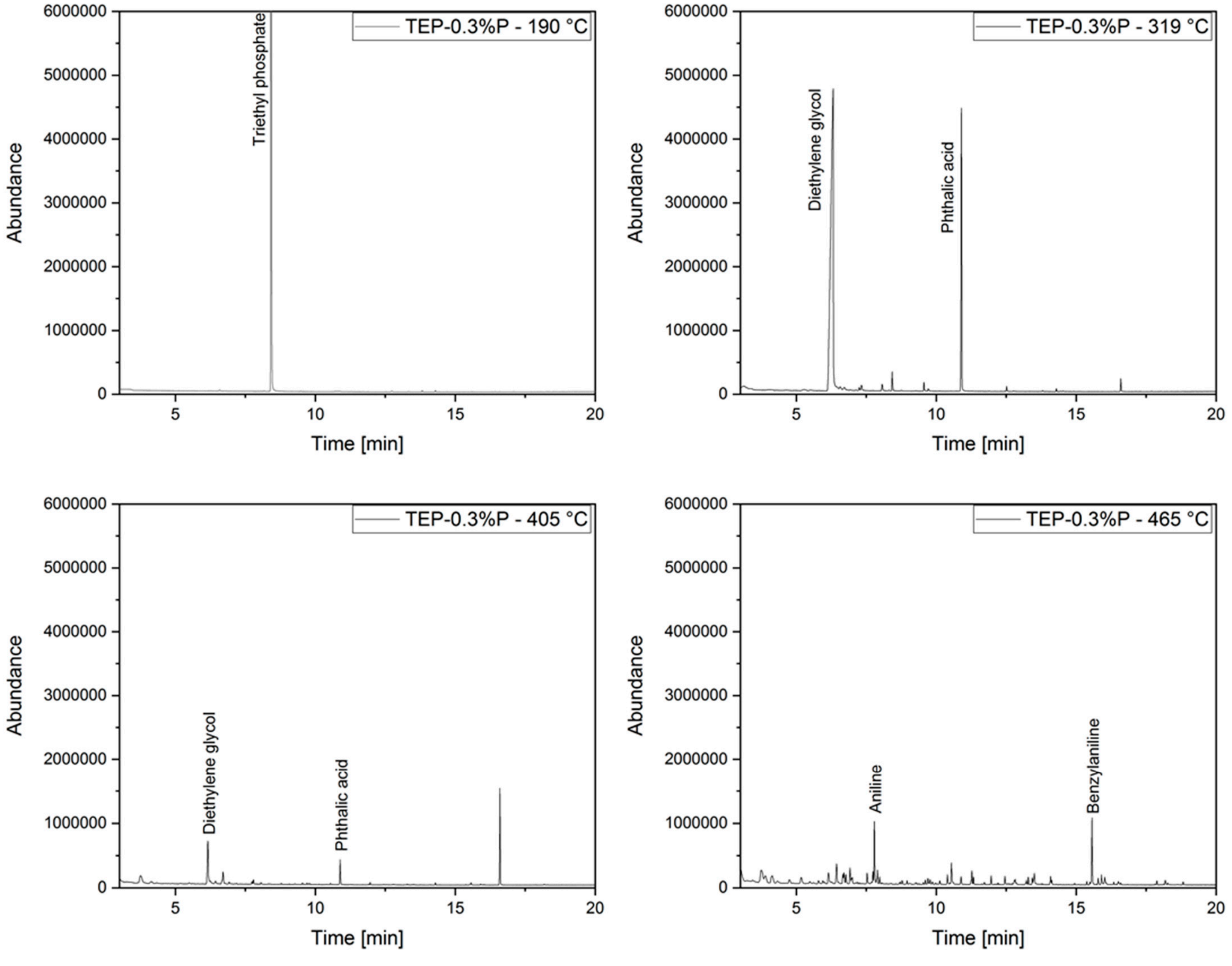

Figure S6. Pyr-GC/MS spectra of **TEP-0.3%P**.

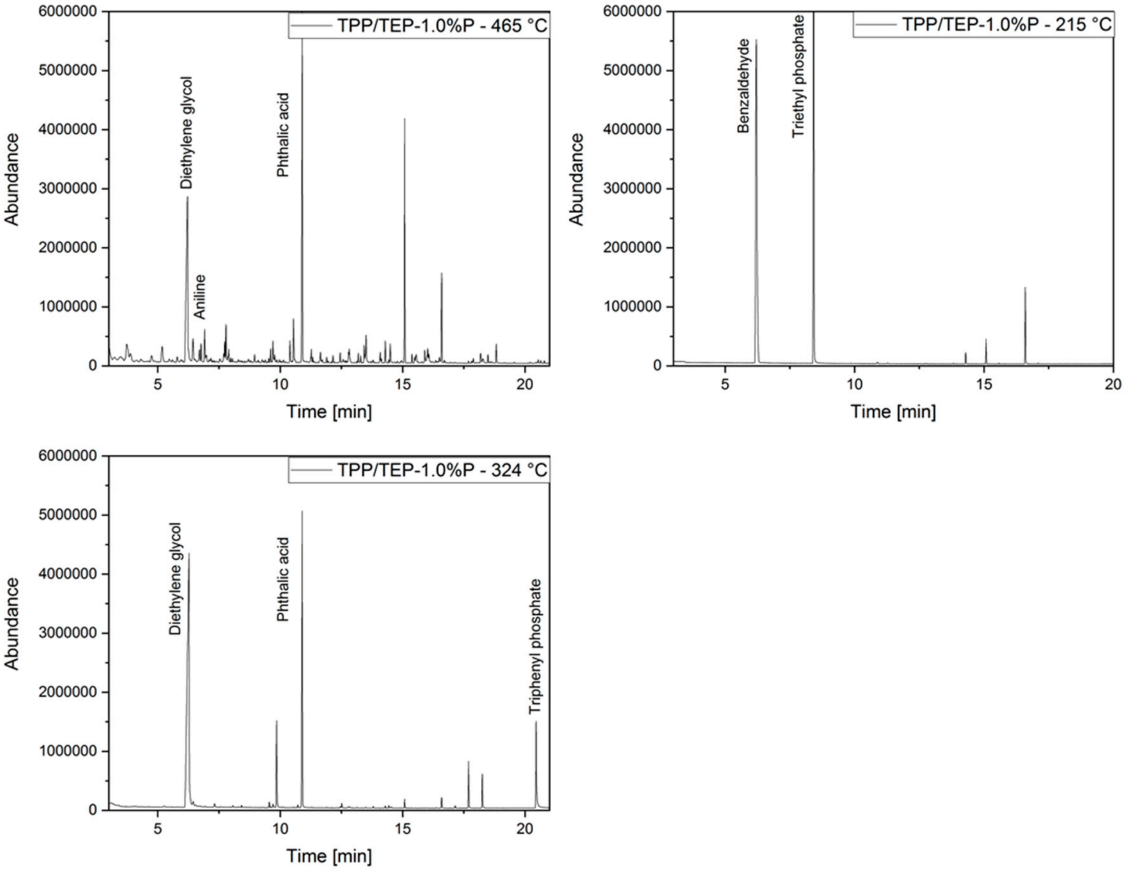

Figure S7. Pyr-GC/MS spectra of **TPP/TEP-1.0%P.**

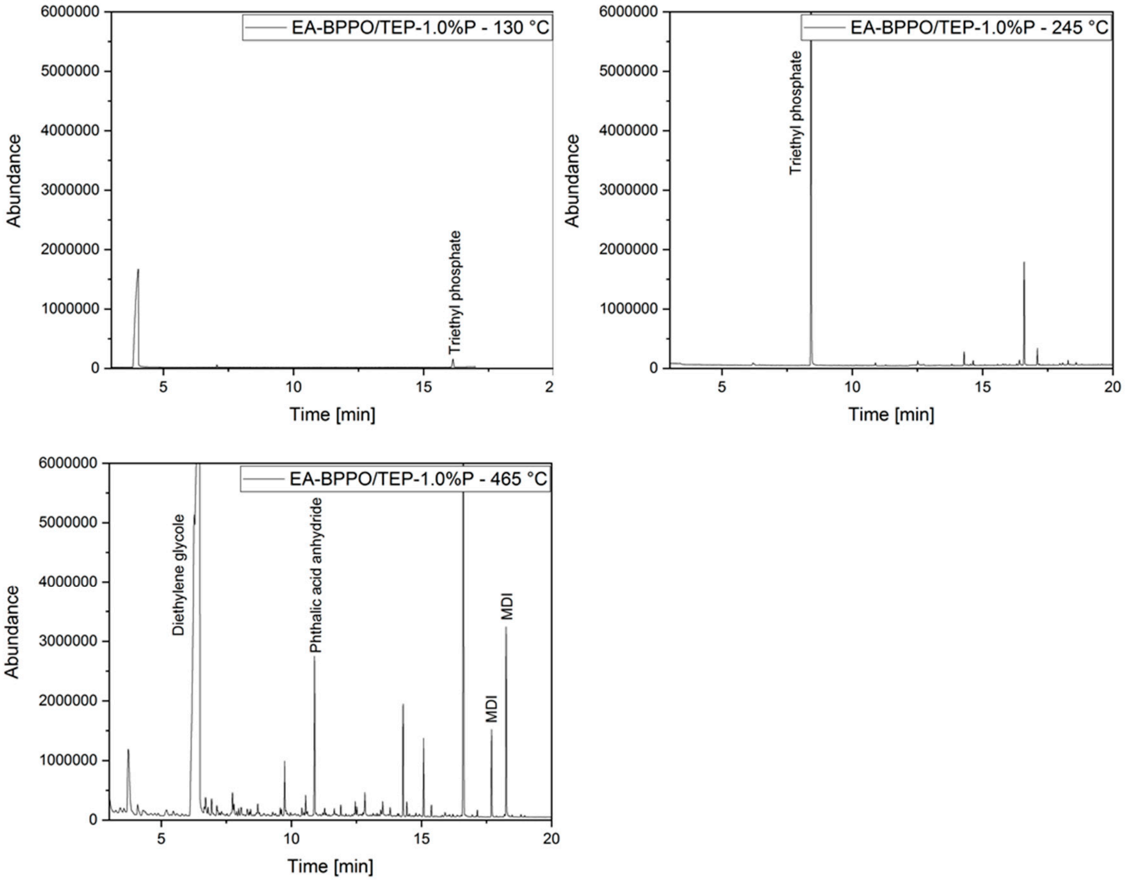

Figure S8. Pyr-GC/MS spectra of **EA-BPPO/TEP-1.0%P.**

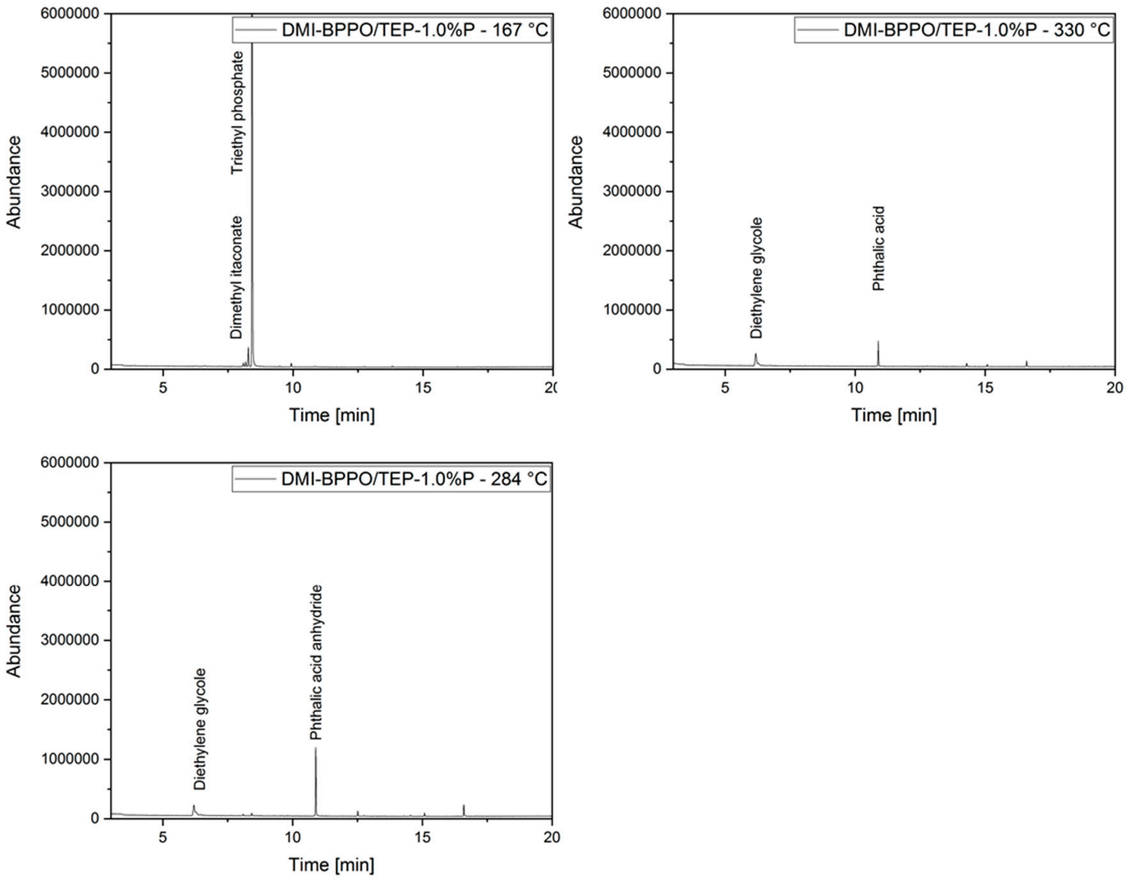

Figure S9. Pyr-GC/MS spectra of **DMI-BPPO/TEP-1.0%P.**

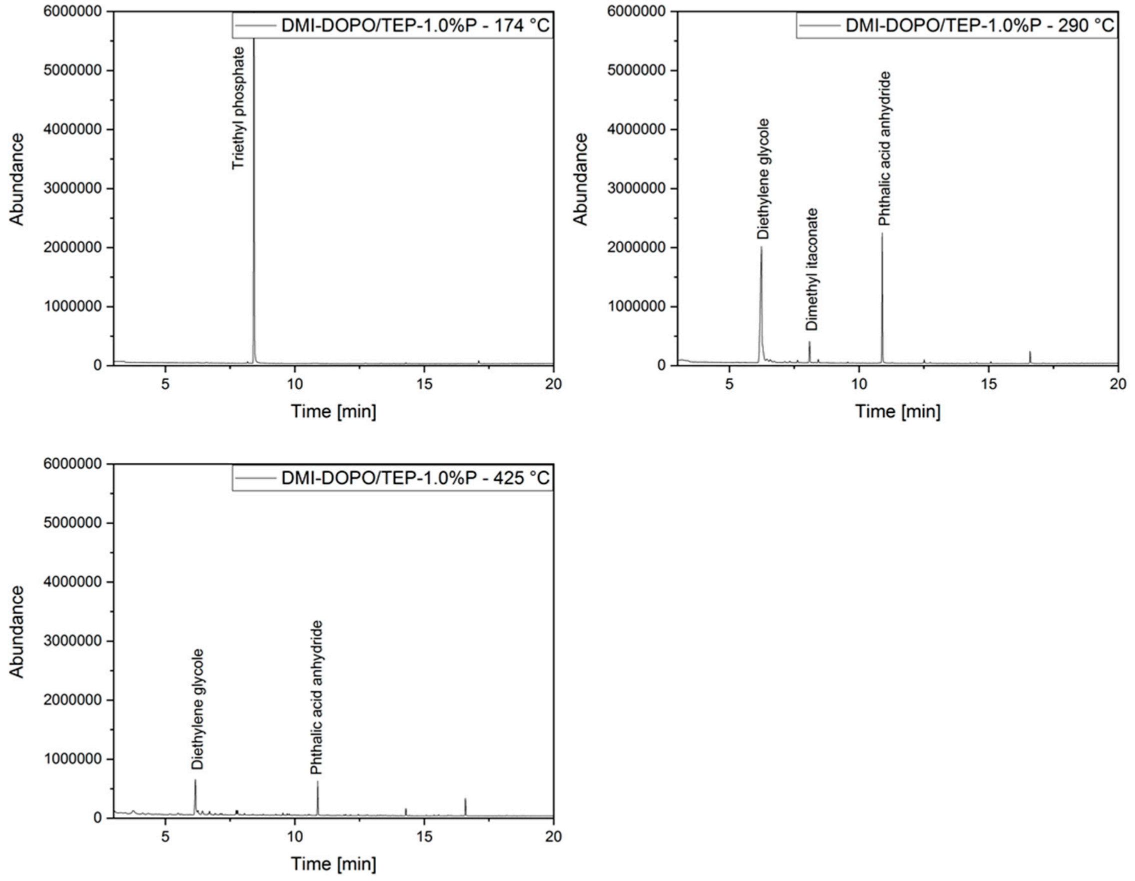

Figure S10. Pyr-GC/MS spectra of **DMI-DOPO/TEP-1.0%P.**

6. Quantitative determination of phosphorus contents

Table S5. Quantitative phosphorus contents of selected foams and after residues after cone calorimetry.

	**P-content [wt%]**	
	**Foam**	**Fire residue**
**TEP-0.3%P**	0.58	0.74
**TPP/TEP-1.0%P**	1.17	2.22
**EA-BPPO/TEP-1.0%P**	1.31	1.54
**MA-BPPO/TEP-1.0%P**	1.15	2.21
**EA-BPPO/TEP-1.3%P**	1.25	1.74

7. Cone calorimetry

Table S6. Complete results of cone calorimeter tests on the PIR foams under study.

Foam	TTI	PHHR	T_{PHHR}	THR	MARHE	Residue	EHC	TSR	CO yield
	s	kW·m^{-2}	s	MJ·m^{-2}	kW·m^{-2}	wt%	MJ·kg^{-1}	m^2·m^{-2}	kg·kg^{-1}
**Ref-0%P**	2 ± 1	233 ± 5	11 ± 2	24 ± 1	172 ± 2	22.3 ± 0.1	2.0 ± 0.1	551 ± 5	0.04 ± 0.01
**TEP-0.3%P**	1 ± 1	178 ± 1	9 ± 1	26 ± 1	128 ± 3	26.7 ± 3.6	2.1 ± 0.1	392 ± 18	0.05 ± 0.01
**TPP-0.7%P**	1 ± 1	205 ± 6	11 ± 1	27 ± 1	145 ± 4	44,7 ± 1,9	2,1 ± 0,1	755 ± 17	0.10 ± 0.01
**TPP/TEP-1.0%P**	1 ± 1	164 ± 1	10 ± 1	20 ± 1	111 ± 4	36,4 ± 2,1	1,8 ± 0,1	324 ± 24	0.08 ± 0.01
**TPP/TEP-1.3%P**	1 ± 1	163 ± 6	10 ± 1	24 ± 1	114 ± 5	27,8 ± 0,9	1,8 ± 0,1	407 ± 59	0.13 ± 0.01
**TPP/TEP-1.5%P**	2 ± 1	162 ± 1	10 ± 1	22 ± 1	112 ± 1	28,6 ± 0,3	1,8 ± 0,1	389 ± 9	0.13 ± 0.01
**MA-BPPO/TEP-1.0%P**	1 ± 1	185 ± 6	10 ± 1	28 ± 1	132 ± 1	28.3 ± 4.6	2.2 ± 0.2	527 ± 28	0.08 ± 0.01
**MA-BPPO/TEP-1.2%P**	1 ± 1	162 ± 4	10 ± 1	23 ± 1	112 ± 6	33.1 ± 1.7	2.0 ± 0.1	421 ± 19	0.07 ± 0.01
**MA-BPPO/TEP-1.4%P**	1 ± 1	167 ± 11	10 ± 1	25 ± 1	114 ± 10	34.4 ± 1.8	1.9 ± 0.1	488 ± 113	0.07 ± 0.01
**EA-BPPO-0.7%P**	1 ± 1	180 ± 13	10 ± 1	26 ± 1	132 ± 1	28,2 ± 0,2	2,0 ± 0,1	501 ± 17	0.08 ± 0.01
**EA-BPPO/TEP-1.0%P**	1 ± 1	168 ± 7	9 ± 1	23 ± 1	121 ± 6	28.6 ± 1.7	1.9 ± 0.1	433 ± 54	0.06 ± 0.01
**EA-BPPO/TEP-1.0%P**	1 ± 1	156 ± 10	9 ± 1	24 ± 1	113 ± 2	29.2 ± 2.5	1.9 ± 0.1	506 ± 25	0.07 ± 0.01
**EA-BPPO/TEP-1.5%P**	1 ± 1	139 ± 7	9 ± 1	27 ± 3	103 ± 6	35.7 ± 0.5	2.1 ± 0.2	398 ± 20	0.07 ± 0.01
**tBuA-BPPO/TEP-1.0%P**	2 ± 1	166 ± 5	10 ± 1	26 ± 1	114 ± 2	27,0 ± 2,2	2,0 ± 0,1	380 ± 72	0.08 ± 0.01
**DMI-BPPO/TEP-1.0%P**	2 ± 1	171 ± 8	9 ± 1	21 ± 2	118 ± 5	33.2 ± 5.2	1.9 ± 0.1	317 ± 32	0.04 ± 0.01
**DMI-DOPO/TEP-1.0%P**	1 ± 1	162 ± 19	10 ± 1	24 ± 2	108 ± 17	29.4 ± 3.6	1.9 ± 0.1	371 ± 66	0.05 ± 0.01
**HQ-BPPO/TEP-1.0%P**	1 ± 1	374 ± 7	13 ± 1	29 ± 8	266 ± 8	31,7 ± 15,6	1,7 ± 0,1	1228 ± 285	0.09 ± 0.01
**HQ-DOPO/TEP-1.0%P**	2 ± 1	134 ± 6	13 ± 1	22 ± 1	101 ± 3	38,0 ± 2,0	1,8 ± 1	503 ± 77	0.09 ± 0.01
